# Curcumin enhances developmental competence and ameliorates heat stress in *in vitro* buffalo *(Bubalus bubalis*) embryos

**DOI:** 10.14202/vetworld.2024.2433-2442

**Published:** 2024-11-05

**Authors:** Ritika Ritika, Sudha Saini, Shavi Shavi, P. N. Ramesh, Naresh L. Selokar, Ashutosh Ludri, Manoj Kumar Singh

**Affiliations:** 1 Animal Biotechnology Division, ICAR-National Dairy Research Institute, Karnal, Haryana, India; 2 Animal Physiology Division, ICAR-National Dairy Research Institute, Karnal, Haryana, India

**Keywords:** antioxidant, buffalo, curcumin, embryo, heat stress

## Abstract

**Background and Aim:**

Buffalo is the principal dairy animal and plays a major role in the economic growth of the dairy industry, contributing nearly 50% of the country’s milk production. The Buffalo core body temperature is typically 38.5°C, but it can rise to 41.5°C in the summer, causing heat stress, which leads to the generation of reactive oxygen species or oxidative stress and affects the reproductive physiology of animals. Curcumin acts as an antioxidant, improves cellular development, and combats the effect of heat stress on *in vitro-*produced embryos. This study aimed to examine the impact of curcumin on developmental competence and the expression of important genes under normal and heat-stressed conditions during *in vitro* embryo production in buffalo.

**Materials and Methods:**

Group-1: All embryo production steps (i.e., *in vitro* maturation [IVM], *in vitro* fertilization [IVF], and *in vitro* culture [IVC]) were conducted at 38.5°C. The presumed zygotes were cultured in media supplemented with different concentrations of curcumin, that is, 0 μM, 5 μM, and 10 μM of curcumin. Group-2: All embryo production steps (i.e., IVM, IVF, and IVC) were carried out at 38.5°C. The presumed zygotes were cultured in media supplemented with different concentrations of curcumin, that is, 0 μM, 5 μM, and 10 μM of curcumin, but the early cleaved embryos were exposed to heat stress (39.5°C) for 2 h after 48 h of IVF and then cultured at 38.5°C for embryo production.

**Results:**

Blastocyst production was 16.63 ± 1.49%, 21.46 ± 0.67%, and 6.50 ± 1.17% at control, 5 μM and 10 μM of curcumin at 38.5°C, respectively, whereas at 39.5°C, it was 8.59 ± 1.20%, 15.21 ± 1.31%, and 3.03 ± 1.20% at control, 5 μM and 10 μM curcumin, respectively. The blastocyst rate was found to be significantly higher (p < 0.05) at 5 μM curcumin compared with the control or 10 μM at 38.5°C and 39.5°C. The antioxidant, antiapoptotic, and pluripotency-related genes exhibited higher (p < 0.05) expression in the presence of 5 μM curcumin compared to 10 μM or control at both temperatures.

**Conclusion:**

Curcumin supplementation in embryo culture media effectively enhances embryo production *in vitro* and mitigates the adverse effects of heat stress.

## Introduction

Escalating global temperatures, combined with the global increase in the number of production animals and intensification of agriculture [1], including emerging economies, has resulted in heat stress, which has become an important challenge faced by the global dairy industry [2]. The average global temperature is expected to increase by about 2°F–13°F (1°C–7°C) at the end of the century. In the scenario of global climatic change, different environmental stresses pose severe threats to animal production globally. Heat stress is a set of conditions that occur when an animal is overexposed to high environmental temperatures or overexerts itself, resulting in an inability to dissipate sufficient heat to maintain homeothermy [3].

In India, buffalo is the primary dairy animal, accounting for more than half of the country’s milk production. The buffalo’s core body temperature is typically 38.5°C but can rise to 41.5°C when exposed to solar radiation during the summer. Although buffaloes are superbly adapted to hot and humid climatic conditions, selection for this tolerance has traditionally resulted in impaired productive and reproductive performance [4]. The disruption of reproduction during heat stress is caused by the failure of an animal to tolerate heat stress, leading to a rise in temperature above its regulatory limit, which could compromise the functioning of germ cells and the viability of an early-developing embryo [5].

The ovarian pool of oocytes is damaged by heat stress during the early stages of folliculogenesis, as indicated by reports that ovarian recovery from summer thermal stress requires a period of two to three estrous cycles before competent oocytes are available. The viability of oocytes collected from heat-stressed cattle that develop to the blastocyst stage following *in vitro* fertilization (IVF) is compromised in normal cycling and repeat breeding cattle [6]. A close correlation was observed between the seasons of the year and the percentage of good-quality oocytes. Buffalo oocytes recovered and matured better in the spring than summer [5].

The percentage of embryos reaching the blastocyst stage was reduced during pre-implantation development due to exposure to heat stress in the maternal body [7]. It has been reported during many studies that embryo loss mainly occurs at the pre-implantation stages; thus, pre-implantation embryos have been distinguished as stress-sensitive. The heat sensitivity of embryos is stage-dependent. Thus, early-stage embryos, such as 1–8-cell stage embryos, are more susceptible to elevated temperatures than advanced-stage embryos, such as morulae or blastocysts [8]. Oxidative stress, mediated by oxygen (O_2_)-derived free radicals (also called reactive oxygen species [ROS]), is often a frequent state that affects nearly all living organisms due to suboptimal environmental conditions. Heat stress other than *in vitro* culture (IVC) conditions is considered to cause oxidative stress in oocytes by generating a superoxide anion (O^2-^) or hydrogen peroxide (H_2_O_2_). ROS levels may be elevated endogenously during many physiological and reproductive processes, including ovulation [9]. To scale back oxidative stress, antioxidant supplementation can potentially decrease damage caused by ROS, thereby preserving the number and quality of oocytes within the ovary. The incidence of oxidative stress-mediated by ROS was found to have a negative effect on the female reproductive system, leading to infertility. Antioxidants, both enzymatic and non-enzymatic, provide the defense against thermal stress-induced oxidative stress [10]. Concerning the role of IVF, the outcomes of various antioxidant supplementations on the standard and cryotolerant of *in vitro*-produced embryos, along with the positive effects on the *in vitro* maturation (IVM) of oocytes and early embryonic development, were assessed using many experimental models.

Curcumin is a yellow substance obtained from *Curcuma longa* plants, which belong to the *Zingiberaceae* ginger family [11]. Curcumin acts as an antioxidant because it scavenges reactive O_2_ and nitrogen species and induces cytoprotective enzymes such as glutathione-S-transferase, γ-glutamyl cysteine ligase, and heme oxygenase-1 [12]. It can scavenge hydrogen peroxide, peroxyl radicals, O^2-^, hydroxyl radicals (OH^-^), singlet O_2_, nitric oxide, and peroxynitrite anion [13]. It has been revealed that curcumin induces endogenous antioxidant defense systems by modulating transcription factors such as nuclear factor (erythroid-derived 2)-like 2, activator protein-1, and nuclear factor kappa B [14]. Modifying feeding management by adding curcumin as a feed supplement improves animals’ overall reproductive and behavioral physiology by combating the effects of heat stress [1].

Looking at the antioxidant properties of curcumin, this is the first study focused on the effects of heat stress and curcumin supplementation in IVC media on buffalo embryos production, their viability, and enzymatic defense system.

## Materials and Methods

### Ethical approval

Buffalo oocytes were obtained from slaughterhouse-based ovaries, and live animals were not used. For this work, ethical approval was granted (Approval No. 43-IAEC-18-36) by the Ethics Committee of the ICAR-National Dairy Research Institute, Karnal (India).

### Study period and location

This study was conducted from October 2022 to September 2023 at ICAR-National Dairy Research Institute, Karnal.

### Reagents and media

The culture media used in the present study included tissue culture medium (TCM-199), Dulbecco’s phosphate-buffered saline, and supplements, including fatty acid-free bovine serum albumin (FAF-BSA), curcumin, follicular stimulating hormone, β-estradiol, mineral oil, and antibiotics such as gentamicin, penicillin, and streptomycin. All chemicals were purchased from Sigma-Aldrich Chemicals, St. Louis, MA, USA. The majority of chemicals used were embryo culture tested or cell culture grade. Fetal bovine serum (FBS) was obtained from Gibco, USA. Research vitro cleave (RVCL) media for embryo culture were obtained from William A Cook, Brisbane, Australia. All chemicals used to prepare Bracket Oliphant and modified Charles Rosenkrans medium were purchased from Sigma Chemical Co., MA, USA, otherwise specified. Disposable plasticware was purchased from Nunc (Roskilde, Denmark), whereas the 0.22-μm filters were purchased from Millipore Corp., Bedford, MA, USA.

### *In vitro* embryo production

Buffalo ovaries were collected from a local slaughterhouse and washed 3 times with warm (37°C) isotonic saline (penicillin 400 IU/mL and streptomycin 500 μg/mL). An 18-gauge needle was used to aspirate cumulus-oocyte complexes (COCs) from follicles with a diameter of 2 mm–8 mm in aspiration media (TCM-199, 0.3% BSA, L-glutamine and 50 μg/mL gentamicin sulfate). The aspirated COCs were washed with a washing medium (TCM199 + 10% FBS + 0.81 mM sodium pyruvate + L-glutamine + 50 μg/mL gentamicin sulfate). After being washed 4–6 times, IVM medium (TCM199 + 10% FBS + 1 μg/mL estradiol-17β + 5 μg/mL porcine follicle stimulating hormone (pFSH)+ 0.81 mM sodium pyruvate + 0.68 mM L-glutamine + 50 μg/mL gentamicin sulfate) was used to culture the groups of COCs (15–20 COCs/100 μL drop), which were then covered with sterile mineral oil and incubated for 24 h at 38.5°C in their respective treatment and control groups in a humid incubator (carbon dioxide [CO_2_] 5%; relative humidity >95%). After 24 h of incubation, IVF wash medium, (Bracket and Oliphant) medium containing 10 μg/mL heparin, 137.0 μg/mL sodium pyruvate, and 1.942 mg/mL caffeine sodium benzoate) was used to wash the expanded COCs, which were then transferred 15–20 oocytes/50 μL drops of IVF medium (IVF wash medium + 1% FAF-BSA). Sperms were prepared for fertilization as described by Singh *et al*. [15], and processed sperms were coincubated with oocytes for 16 h–18 h. After incubation, the spermatozoa were removed, and the presumptive zygotes were then cultured in 100 μL droplets of IVC medium (RVCL™, Cook Medical, Australia) for 8-day post-insemination at 38.5°C in a humidified CO_2_ incubator (5% CO_2_ in air and > 95% relative humidity). The cleavage rate was checked on day 2 post-insemination, and the percentages of oocytes that developed to 4, 8, and 16 cell stages, morulae, and blastocyst stages were recorded on days 3, 4, 5, and 8 post-insemination.

### Quantitative real-time polymerase chain reaction (PCR)

PCR was performed to amplify target and reference genes using a real-time thermocycler (Bio-Rad CFX96™, C1000™, (California, USA) with Master SYBR Green I mix (Fermentas, USA). Total RNA was isolated from blastocysts (n *=* 10 each) using an RNAqueous Micro Kit (Ambion Inc., USA) according to the manufacturer’s instructions. The concentration and purity of RNA were determined using Nanoquant (Teccan, Austria). Following DNase treatment, complementary DNA (cDNA) was prepared using a Superscript III first-strand cDNA synthesis kit (Invitrogen, USA) and stored at −80°C until used for quantitative PCR (qPCR). PCR amplification was performed using one cycle of initial denaturation at 95°C for 3 min, 35 cycles of denaturation (95°C for 15 s), primer annealing temperature for 30 s (Table-1), and extension (72°C for 30 s). Glyceraldehyde-3-phosphate dehydrogenase expression was taken as an endogenous reference. In negative controls, nuclease-free water was used as the template. After amplification, cycle threshold (Ct) values of the control and experimental groups with reference genes were taken to calculate fold changes in target gene expression.

**Table-1:** Primers used in gene expression study.

Gene	Primer sequence	Size	Annealing temperature (°C)	Accession No.
Antioxidant-related genes				
*SOD2*	F-AATCTGAGCCCTAACGGTGG R-CAATCTGTAAGCGTCCCTGC	175	60	XM_018053428.1
*SOD3*	F-AGGCCTTCTTCCACCTTGAG R-GAAGTTGCCAAAGTCGCCC	167	60	XM_018049136.1
*GPX1*	F-GCTCTCATGACCGACCCTAA R-GGGACAGCAGGGTTTCAATG	169	60	XM_005695962.3
*GPX2*	F-TCCTTCTACGACCTCAGTGC R-TTGCAAGGGAAGCCAAGAAC	188	60	XM_005685982.3
Heat-shock -related genes				
*HSP10*	F-GAGTATTAGTTGAAAGAAGTGCG R-ACTTTGGTGCCTCCATATTCTG	199	60	NM_174346.2
*HSP60*	F-ACTGGCTCCTCATCTCACTC R-TGTTCAATAATCACTGTCCTTCC	147	60	NM_001166610.1
Apoptosis-related genes				
*BAD*	F- CCAGAGCATGTTCCAGATCC R- GTTAGCCAGTGCTTGCTGAG	125	60	XM_005699970
*BAX*	F-CCTTTTGCTTCAGGGTTTCA R- CGCTTCAGACACTCGCTCA	123	60	NM_001191220.1
*BCL-XL*	F-TTGTGGCCTTTTTCTCCTTC R-GATCCAAGGCTCTAGGTGGT	128	60	ENSBTAT00000008572
*P53*	F-GGAAGAATCACAGGCAGAACTC R-ACTTCATTCGGACATTCATCCA	176	60	AB571118.1
Pluripotency-related genes				
*OCT4*	F-GTTCTCTTTGGAAAGGTGTTC R-ACACTCGGACCACGTCTTTC	214	60	AF022987
*SOX2*	F- ACCAGCTCGCAGACCTACAT R- GGTAGTGCTGGGACATGTGA	265	60	NM_001105463.2
*NANOG*	F- ACTTTCCAACATCTTGAACCTC R- GTATGCCATTGCTATTTCTCGG	116	60	NM_001025344.1
*C-MYC*	F-ACAGGCAGCTGGATGAGACT R-TGTGGGTGAAGGAGACTCTG	230	60	AJ812564
*GAPDH*	F- TCAAAGAAGGTGGTGAAGCAG R- CCCAGCATCGAAGGTAGAAG	123	60	NM_001034034.2

*SOD*=Superoxide dismutase, *GPX1*=Glutathione peroxidase1, *BAX*=BCL2-associated X, *BCL-XL*=B-cell lymphoma-extra large, *HSP*=Heat shock proteins, *GAPDH*=Glyceraldehyde-3-phosphate dehydrogenase

### Experimental design

Group 1: All embryo production steps (i.e., IVM, IVF, and IVC) were carried out at 38.5°C. The presumed zygotes were cultured in media supplemented with different concentrations of curcumin, that is, 0 μM, 5 μM, and 10 μM of curcumin. Group 2: All embryo production steps (i.e., IVM, IVF, and except IVC) were carried out at 38.5°C. Curcumin was supplemented with various concentrations, that is, 0 μM, 5 μM, and 10 μM during IVC. The early embryos were exposed to heat stress for 2 h at 39.5°C after 48 h of IVF and then transferred to 38.5°C. cDNA was synthesized from RNA isolated from produced embryos following the manufacturer’s instructions using Superscript III, a first-strand cDNA synthesis kit (Invitrogen). Quantitative gene expression was compared within and between groups at the blastocyst stage.

### Statistical analysis

The data were analyzed using SYSTAT 6.0 (SPSS Inc. Chicago, IL, USA). Differences among means were analyzed by a one-way analysis of variance followed by Fisher’s least significant difference test. Significance was determined at p < 0.05.

## Results

### Effect of curcumin on the developmental competence of *in vitro* pre-implantation embryos

**Figure-1:**
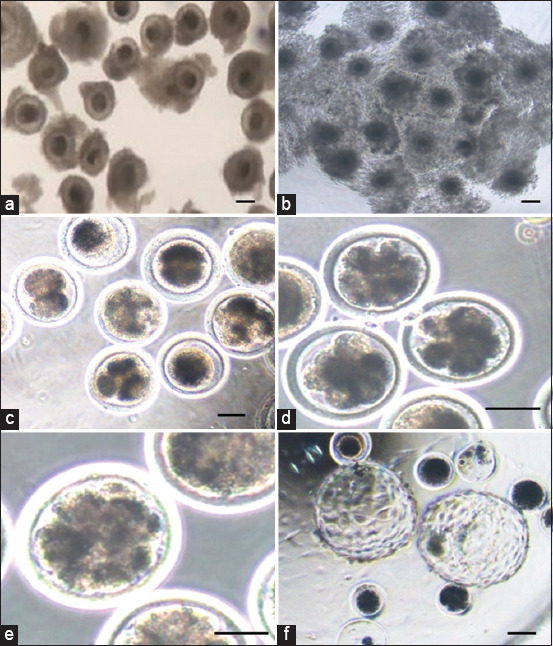
Production of buffalo embryos *in vitro*: (a) Immature oocytes, (b) Mature *in vitro* oocytes, (c) 2–4-cell, (d) 8–16 cells, (e) morula, and (f) hatched blastocysts. Scale bar = 50 μm.

Developmental competence, which included cleavage rate and oocyte percentage that developed into 4-cell, 8-16 cell, morulae, and blastocysts, was compared between both the groups, that is, control and experimental groups. The embryonic stages like 2-cell, 4-cell, 8-16 cell stages, and morulae were significantly higher (p < 0.05) at 5 μM concentration, as compared to 10 μM concentration (Table-2). The percentage of blastocyst formation was 16.63 ± 1.49, 21.46 ± 0.67, and 6.50 ± 1.17% for the control, 5 μM and 10 μM concentration of curcumin, respectively. Similarly, the rate of blastocyst formation is significantly higher (p < 0.05) at 5 μM concentration, as compared to control and 10 μM concentration in non-stressed condition (Figure-1). The results illustrated that curcumin supplementation at a suitable concentration (5 μM) can enhance the developmental competence of *in vitro* fertilized buffalo oocytes (Table-2).

**Table-2:** Developmental competence of embryos cultured in media supplemented with different concentrations of curcumin.

Oocyte/embryonic stages	Curcumin concentration		

	0 μM	5 μM	10 μM
Oocyte (n)	226	245	239
2-cell % (n)	58.75 ± 2.25^a^ (135)	64.11 ± 1.15^b^ (156)	44.66 ± 4.28^c^ (113)
4-cell % (n)	48.86 ± 2.32^a^ (112)	52.80 ± 2.04^a^ (128)	33.09 ± 2.31^b^ (82)
8–16 cell % (n)	39.03 ± 3.50^a^ (90)	45.58 ± 1.47^b^ (111)	27.24 ± 2.36^c^ (68)
Morula % (n)	27.63 ± 0.97^a^ (63)	29.30 ± 0.95^a^ (72)	20.92 ± 2.39^b^ (39)
Blastocyst % (n)	16.63 ± 1.49^a^ (39)	21.46 ± 0.67^b^ (52)	6.50 ± 1.17^c^ (17)

Data generated from three trials. Values are expressed as mean ± standard error of the mean. Values with different superscripts within the same row differ significantly (p < 0.05)

### Effect of curcumin on the development rate of heat-stressed pre-implantation embryos

Curcumin doses were added to pre-implantation embryos at the time of IVC, and the temperature was increased to 39.5°C (Table-3). After which development competence, that is, cleavage rate, 4-cell, 8–16 cell, morulae, and blastocyst percentage) in the treatment groups was compared with the control. The embryonic stages like 2-cell, 4-cell, 8-16 cell stages, and morulae were significantly higher (p < 0.05) at 5 μM concentration, as compared to control and 10 μM concentration (Table-3). The percentage of blastocyst formation was 8.59 ± 1.20, 15.21 ± 1.31, and 3.03 ± 1.20 at control, 5 μM and 10 μM concentrations of curcumin, respectively (Figure-1). Likewise, the rate of blastocyst formation is significantly higher (p < 0.05) at 5 μM concentration, as compared to control and 10 μM concentration (Table-3).

**Table-3:** Developmental competence of heat-stressed embryos cultured in media supplemented with different concentrations of curcumin.

Oocyte/embryonic stages	Curcumin concentration		

	0 μM (Control)	5 μM	10 μM
Oocyte (n)	212	199	209
2-cell % (n)	47.79 ± 4.30^a^ (103)	57.03 ± 2.50^b^ (114)	37.54 ± 0.83^c^ (77)
4-cell % (n)	31.39 ± 0.70^a^ (67)	50.39 ± 2.96^b^ (101)	26.99 ± 0.52^c^ (56)
8–16 cell % (n)	23.21 ± 2.76^a^ (51)	35.25 ± 1.74^b^ (71)	17.81 ± 2.30^c^ (39)
Morula % (n)	14.62 ± 1.60^a^ (32)	23.56 ± 1.47^b^ (46)	9.73 ± 1.04^c^ (21)
Blastocyst % (n)	8.59 ± 1.20^a^ (19)	15.21 ± 1.31^b^ (30)	3.03 ± 1.20^c^ (6)

Data generated from three trials. Values are expressed as mean ± standard error of the mean. Values with different superscripts within the same row differ significantly (p < 0.05)

### Gene expression in *in vitro* blastocysts

#### Genes related to antioxidant activity

**Figure-2:**
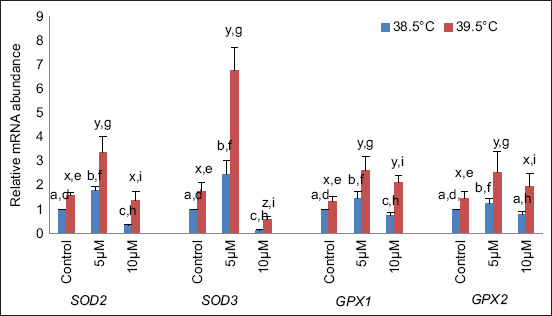
Relative messenger RNA abundances of antioxidant-related genes at different temperatures. Bars with different superscripts differ significantly (p < 0.05) between two different temperatures (a, b, and c: indicate variations within 38.5°C; x, y, and z: indicate variations within 39.5°C and d, e, f, g, h, and i: indicate the variations between these two temperatures).

It was revealed that the relative messenger RNA (mRNA) abundance of antioxidant genes superoxide dismutase (*SOD2* and *SOD3*) was significantly (p < 0.05) higher in *in vitro* buffalo blastocysts produced in culture media supplemented with curcumin (5 μM) and significantly (p < 0.05) lower at 10 μM curcumin than in control (no curcumin) at 38.5°C. At elevated temperature (39.5°C), *SOD2* expression was significantly (p < 0.05) higher at 5 μM curcumin. It non-significantly changed at 10 μM compared with the control. In contrast, in the case of *SOD3*, expression was significantly (p < 0.05) higher at 5 μM and significantly (p < 0.05) lower at 10 μM compared with the control. The expression of both genes (*SOD2* and *SOD3*) was significantly (p < 0.05) up-regulated in blastocysts when temperature was increased at all concentrations (Figure-2). Other antioxidant-related genes glutathione peroxidase (*GPX1* and *GPX2*) were significantly (p < 0.05) higher at 5 μM curcumin supplementation in culture media, and at 10 μM curcumin supplementation, *GPX1* expression was significantly (p < 0.05) low, while *GPX2* was not significantly affected with respect to control (no curcumin) at 38.5°C. At elevated temperature (39.5°C), *GPX1* expression was significantly (p < 0.05) higher at 5 μM and 10 μM curcumin compared with the control, whereas in the case of *GPX2*, expression was significantly (p < 0.05) higher at 5 μM and non-significantly increased at 10 μM compared with the control. The expression of both genes was significantly (p < 0.05) up-regulated at all concentrations at elevated temperatures compared with 38.5°C (Figure-2).

#### Genes related to heat shock

**Figure-3:**
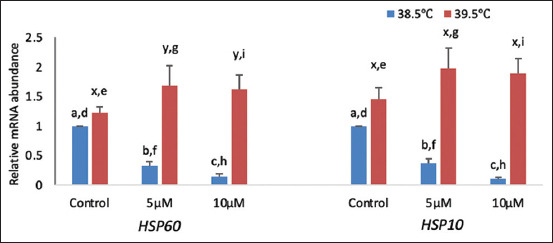
Relative messenger RNA abundances of heat-shock-related genes at different temperatures. Bars with different superscripts differ significantly (p < 0.05) between two different temperatures (a, b, and c: indicate variations within 38.5°C; x, y, and z: indicate variations within 39.5°C and d, e, f, g, h, and i: indicate the variations between these two temperatures).

The effects of elevated temperature during maturation on mRNA expression of heat shock-related genes (heat shock protein [*HSP*]*10* and *HSP60*) mRNA expression are depicted in Figure-3. Results showed that at 38.5°C, the relative mRNA abundance of heat shock genes (*HSP60* and *HSP10*) was significantly (p < 0.05) lower in buffalo blastocysts fed curcumin (5 μM and10 μM) curcumin supplementation as compared to the control. At elevated temperature (39.5°C), HSP60 expression was significantly (p < 0.05) higher at 5 μM and 10 μM curcumin compared with the control, but at 10μM curcumin concentration, gene expression did not vary significantly (p < 0.05) with respect to 5 μM curcumin; *HSP10* expression at 5 μM and 10 μM curcumin concentration did not significantly (p < 0.05) vary within itself as well as compared with the control. The expression of both genes (*HSP60* and *HSP10*) was significantly (p < 0.05) up-regulated in blastocysts in both the presence and control at elevated temperatures (Figure-3).

#### Genes related to apoptotic activity

**Figure-4:**
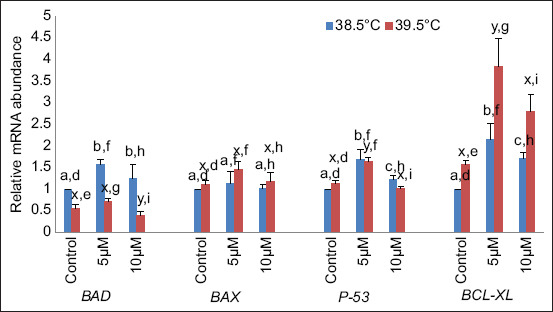
Relative messenger RNA abundances of apoptosis-related genes at different temperatures. Bars with different superscripts differ significantly (p < 0.05) between two different temperatures (a, b, and c: indicate variations within 38.5°C; x, y, and z: indicate variations within 39.5°C and d, e, f, g, h, and i: indicate the variations between these two temperatures).

At 38.5°C, the relative mRNA abundance of the proapoptotic gene *BAD* was significantly (p < 0.05) higher in buffalo blastocysts produced in culture media supplemented with curcumin 5 μM and 10 μM curcumin compared to control, but non-significant (p < 0.05) between these two concentrations. At the same temperature, BCL2-associated X (*BAX*) expression did not differ significantly (p < 0.05) at 5 μM and 10 μM curcumin compared with the control. At 39.5°C, the expression of *BAD* was non-significant (p < 0.05) different at 5 μM compared with the control, but at 10 μM curcumin supplementation, *BAD* expression was significantly lower compared with the control. In the case of *BAX* at 39.5°C, the relative mRNA expression did not vary significantly (p < 0.05) at both concentrations (5 μM and 10 μM) as compared to the control. Antiapoptotic genes (*P53* and B-cell lymphoma-extra large [*BCL-XL*]) were significantly (p < 0.05) higher at 5 μM and 10 μM curcumin supplementation in culture media compared with the control at 38.5°C. At elevated temperature (39.5°C), P53 expression was significantly (p < 0.05) higher at 5 μM but did not differ significantly (p < 0.05) at 10 μM compared with the control, whereas in the case of *BCL-XL* expression, it was significantly (p < 0.05) higher at 5 μM and non-significantly increased at 10 μM compared with the control. The expression of both antiapoptotic genes was significantly (p < 0.05) up-regulated at all concentrations at elevated temperatures compared with 38.5°C (Figure-4).

#### Genes related to pluripotent activity

**Figure-5:**
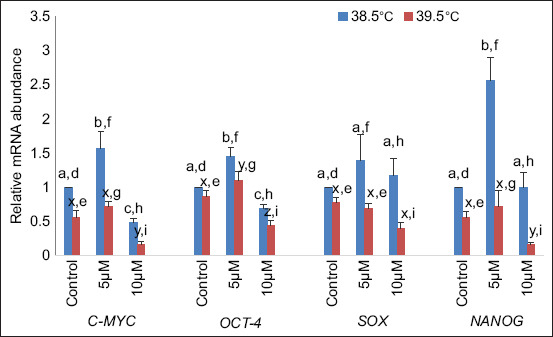
Relative messenger RNA abundances of pluripotency-related genes at different temperatures. Bars with different superscripts differ significantly (p < 0.05) between two different temperatures (a, b, and c: indicate variations within 38.5°C; x, y, and z: indicate variations within 39.5°C and d, e, f, g, h, and i: indicate the variations between these two temperatures).

In the current study, it was observed that at 38.5°C, the relative mRNA abundance of pluripotent genes (*C-MYC* and *OCT4*) was significantly (p < 0.05) higher in buffalo blastocysts produced in culture media supplemented with curcumin (5 μM) and significantly (p < 0.05) lower at 10 μM curcumin supplementation than in the control. At higher temperature (39.5°C), C*-MYC* expression did not differ significantly (p < 0.05) at 5 μM curcumin and significantly lower (p < 0.05) at 10 μM curcumin compared with the control, whereas OCT4 expression was significantly (p < 0.05) higher at 5 μM and significantly (p < 0.05) lower at 10 μM curcumin compared to control). Whereas, *SOX* expression was non-significantly (p < 0.05) different at 5 μM and 10 μM curcumin supplementation in culture media with respect to control at 38.5°C as well as 39.5°C. In the case of *NANOG*, at 38.5°C, the relative mRNA abundance was significantly (p < 0.05) higher during the supplementation of curcumin (5 μM) and non-significant (p < 0.05) at 10 μM curcumin supplementation compared with the control. At 39.5°C, *NANOG* expression was non-significant (p < 0.05) at 5 μM compared with the control, but at 10 μM curcumin supplementation, *NANOG* expression was significantly lower (p < 0.05) as compared to control. The expression of all pluripotency genes was significantly (p < 0.05) down-regulated at both concentrations at elevated temperatures compared with 38.5°C (Figure-5)

## Discussion

Although buffalo is India’s mainstay of dairy agriculture, information on combating the direct effects of heat stress on buffalo oocytes and embryonic development and ameliorating these effects is scant [16]. Heat stress associated with O_2_-derived free radicals negatively affects reproductive performance and leads to decreased implantation and increased early embryonic loss [17]. Therefore, improving the thermal resistance of embryos is essential for efficient animal production. Tripathi *et al*. [10] and Chauhan *et al*. [18] have demonstrated that the balanced presence of antioxidants and ROS during *in vitro* embryo production media can be beneficial for early embryonic development. The natural antioxidant property of curcumin is widely utilized in various biological processes where it potentially reduces lipid peroxides and augments the activity of antioxidant enzymes such as *SOD*, catalase, and glutathione reductase, which have ROS scavenging properties [10]. It has already been reported that the culture media used for *in vitro* embryo production and the gaseous environment provided are always sub-optimal and sometimes even beyond control. Culture media contain several supplements for embryonic growth, but the use of antioxidants plays a crucial role in maintaining the redox potential of cultured cells/embryos [19]. In the present study, we used curcumin as an antioxidant in culture media to combat heat stress in embryos and observed changes in gene expression, which could lead to several possible interventions for improving embryo production efficiency. For this study, we used curcumin at different concentrations and found a positive response regarding the increased embryo production rate, which may be due to the antioxidant properties of curcumin to maintain optimum culture conditions.

To the best of our knowledge, this is the first study on the use of curcumin to combat the effect of heat stress on buffalo embryos, although the effects of heat stress and antioxidants on buffalo oocytes and embryo production have been extensively researched [20]. In this study, we found significantly decreased (p < 0.05) developmental competence of the oocytes when the temperature was increased to 39.5°C from a normal temperature of 38.5°C, which is following the study conducted by Ashraf *et al*. [21], at elevated temperatures, that is, 40.5°C and 41.5°C, the development of oocytes to blastocysts was severely compromised (p < 0.001) and the cleavage rates, blastocyst yield, and mean cell number decreased remarkably (p < 0.001) compared with the control. Similarly, low development competence and a high level of apoptosis was observed when the oocytes were given 2 h of heat shock daily during IVM, fertilization, and culture [22]. As heat stress fastens cellular metabolism, mitochondria are unable to efficiently decrease O_2_, which is in its free radical state, causing oxidative stress by generating O_2_^-^ or H_2_O_2_ [23]. The ROS, which includes O_2_^-^, H_2_O_2_, and OH^-^ have the capacity to react with any molecule and change it oxidatively. Oxidative stress occurs because of an imbalance between the scavenging capacity of antioxidant defense systems and the production of ROS [24]. Chauhan *et al*. [18] reported that the presence or absence of ROS significantly affects major factors, such as growth and development, in normal cells.

To date, there are no reports on curcumin effectively combating heat stress in buffalo *in vitro*-produced embryos. It was reported that different antioxidants, that is, 0.6 mM cysteine, 0.6 mM cysteine + 100 μM cysteamine, 100 IU catalase, or 100 μM mercaptoethanol, which were used for IVC, when supplemented alone or in combination, efficiently support high (p < 0.05) blastocyst production (43.60% to 48.50%) compared with the control (36.45%) in bovines [25]. Similarly, Goel *et al*. [26] reported that the use of insulin-like growth factor I (IGF-I), cysteamine, or both significantly increased the blastocyst production rate (p < 0.05) in comparison to the control group (17%–18% vs. 7%) in caprine. Our study is also following El Hosiny *et al*. [27], where buffalo oocytes mature and cultured in media with different concentrations (0, 25, 50, and 100 μM) of ascorbic acid and found a significant (p < 0.01) increase in the cleavage rate at 25 μM and 50 μM of ascorbic acid compared with the control and 100 μM groups, and the percentage of blastocyst rate was significantly higher in 25 μM (p < 0.01), 50 μM (p < 0.001), and 100 μM (p < 0.05) ascorbic acid groups than the control, hence indicating that the concentration of antioxidant also affects the embryo production rate. It has been reported that after IVF in bovines, blastocyst rates were higher in the quercetin, Vitamin C, resveratrol, cysteamine, or carnitine groups than in the control group (p < 0.05) [28]. Roshan *et al*. [29] reported a significant increase (p < 0.05) in blastocyst production when 50 μM L-ascorbic acid was used in IVM and IVC compared with the control. In addition, heat stress reduced the growth of COC diameters of grades-A (about 50%) and -B (about 40%). 7.5 μM retinol considerably increased (p < 0.01) the expansion in grade-B COCs at 41°C, while 1 nM melatonin and 1.5 g/mL zinc chloride significantly increased the COC diameter at 38.5°C in grade-A COCs. This resulted in significantly higher (p < 0.01) maturation, fertilization, and cleavage rates. In addition to improving maturation outcomes, retinol supplementation is a superior antioxidant for combating the harmful effects of high temperatures [30]. Keeping in view the foregoing discussion, it is evident that the concentrations of curcumin (5 μM, 10 μM) used in the present study at different temperatures (38.5°C and 39.5°C) are relevant to ameliorate the effect of heat stress on the developmental competence of *in vitro* cultured buffalo embryos. Moreover, it is likely that curcumin at a lower concentration 5 μM is more efficient in ameliorating heat stress and subsequent ROS effects in embryos than at a higher concentration, as per the present results.

In the present study at 38.5°C, the relative mRNA abundance of heat-stressed (*HSP10* and *HSP60*) genes and antioxidant-related genes (*SOD2, SOD3, GPX1*, and *GPX2*) was significantly higher (p < 0.05) in blastocysts produced in 5 μM group as compared to control and 10 μM concentration of curcumin group. At 38.5°C, the relative mRNA abundance of pro-apoptotic (*BAD and BAX*) and anti-apoptotic (*P53 and BCL-XL*) genes was significantly lower (p < 0.05) for blastocyst of the 10 μM group as compared to group of 5 μM curcumin. Our results are in agreement with Wang *et al*. [31], who treated HT-29 cells with 10 μM–80 μM curcumin for 24 h and found that curcumin (40 μM–80 μM) decreased the transcriptional *BCL-2/BAX* ratio. The anti-apoptotic proteins *BCL-XL* and *SURVIVIN* were transcriptionally down-regulated significantly (p < 0.05) at higher concentrations. A low O_2_ environment (2% O_2_) reduced the ratio of *BAX/BCL2*, which was further reduced after the addition of Vitamin C, whereas the gene expression level of *BCL2* increased after the addition of Vitamin C under conditions of increasing O_2_ concentrations [32]. Similarly, buffalo oocytes were exposed to an elevated temperature of 39.5°C or 40.5°C for 2 h once daily throughout IVM and IVC [22], except for the heat stress-related gene *HSF1*. Embryos at 39.5°C or 40.5°C had higher relative mRNA abundances of the pro-apoptotic genes *CASPASE-3, BID*, and *BAX* and stress-related genes *HSP 70.1* and *HSP 70.2* at the 8-16-cell and blastocyst stages, respectively (p < 0.05). *BCL-XL* and *MCL-1* expression levels were also greater in embryos at higher temperatures compared with controls at the 8–16-cell and blastocyst stages (p < 0.05). Likewise, a direct impact on bubaline oocytes undergoing IVM during two periods of physiologically appropriate high temperatures (40.5°C and 41.5°C) was reported and found that oocyte maturation to blastocyst formation was severely reduced (p < 0.001) for both, they also reported a significantly higher relative mRNA expression of genes associated with HSP (*HSP 70.1, 70.2, 70.8, 60, 10*, and *HSF1)*, pro-apoptotic (*CASPASES*-3, -7, -8, *BID* and *BAX*), and oxidative stress (inducible nitric oxide synthase). However, the mRNA abundance of genes associated with oxidative stress, glucose transport, developmental competence, and anti-apoptotic activity (*BCL-2, MCL-1, BCL-XL, GLUT1, GLUT3*, and *IGF1R*) was substantially lower (p < 0.05) in the treatment groups than in the control group [21].

To reduce ROS levels in cultured media, embryos were cultured in hypoxic conditions (5% vs. 20% O_2_) and the blastocyst rate was significantly greater (p < 0.05), whereas the percentage of apoptotic-positive cells was much lower (p < 0.05) at low O_2_ concentrations [33, 34]. At various embryonic stages, the expression of the pro-apoptotic genes *BAX* and *BID* was lower (p < 0.05) under 5% O_2_ than under 20% O_2_ concentration. To combat the increased ROS, they supplemented cysteamine and found an increased blastocyst production rate and significantly higher (p < 0.05) relative mRNA abundance of *BCL-XL* and *MCL-1*, whereas *BAX* but not *BID* was lower (p < 0.05). Elamaran *et al*. [33] and Grewal *et al*. [34] support the current investigation in which a similar effect on mRNA expression in pro-apoptotic genes was observed at high temperatures and was further reduced with the help of different doses of curcumin. Our results are also consistent with El-Sayed *et al*. [35], where the oocytes treated with L-ascorbic acid showed a decrease in mRNA expression for all investigated genes (p < 0.05), except *HSP-90* and *HSF1*. The relative mRNA abundance of *MCL1* was considerably decreased in blastocysts formed after the addition of L-ascorbic acid (p < 0.05), whereas that of *BAX* dramatically decreased in contrast to the control group. Similarly, in our study, at 38.5°C, the relative mRNA abundance of the *HSP10* and *HSP60* genes was significantly lower (p < 0.05) for blastocyst of 5 μM and 10 μM groups compared with the control.

Curcumin-treated cells showed a dose-dependent decrease in *HSP60* levels, which was significantly decreased at all concentrations (6, 12.5, and 25 μM) compared with untreated cells [36]. Antioxidants added to culture media counteract the negative effects of heat stress. The proportion of embryos reaching the blastocyst stage was found to be lower when pre-implantation development was subjected to thermal stress in the maternal body. Pre-implantation stage embryos are known to be susceptible to stress, and this is when early embryonic loss occurs. These data suggest that heat stress directly impairs early-stage embryos and reduces their capacity for development. These findings suggest that oxidative stress caused by rising temperatures may be the primary factor impeding proper embryonic development. Preventing this rise in oxidative stress may enable embryos to survive heat stress. Vitamin C induces endogenous antioxidants and *HSPs* to ameliorate heat stress in H9C2 cells, and we observed significantly reduced vacuolation, karyopyknosis, nuclei damage, apoptosis, lactate dehydrogenase activity, ROS, and malondialdehyde levels in the treatment groups, whereas SOD (*SOD2 and SOD3*) activity was increased and mRNA levels of *HSP10*, *HSP60, HSP70*, and others were elevated at (p < 0.01) [37]. These results are consistent with the current study and suggest that the introduction of the natural antioxidant curcumin may prevent ROS production due to heat stress and, thus, heat damage by up-regulating *HSP*.

Antioxidants can reduce the negative consequences of free radicals and, probably, even apoptosis caused by heat stress. The present results are in agreement with Ahmed *et al*. [38], who showed that without supplementation with antioxidants, the expression of *BAD* and *BCL2* increased. *BAD* was downregulated in the melatonin and zinc supplement groups, whereas *BAX* was downregulated in all groups. This suggests that by detoxifying ROS, antioxidants may subsequently reverse the ROS-induced decline in *BCL2* and prevent apoptosis. When turmeric extract was used for 24, 48, and 72 h, it was found that after 24 h, at 20 μg/mL concentration of extract, *OCT4A* and *OCT4B* variant expression decreased at the maximum rate, whereas OCTB1 variants had higher expression [29]. In our findings, the expression levels of *SOX*, *NANOG, OCT4*, and *C-MYC* genes were significantly higher in the presence of 5 μM curcumin at both temperatures compared with the control, revealing that curcumin has a beneficial effect on the quality of embryos.

This study’s findings aid in the development of embryo culture media containing curcumin, which could be beneficial for quality embryo production during heat stress. The *in vivo* studies can provide more insights into the antioxidant properties of curcumin.

## Conclusion

Our findings suggest that culture media and supplements, particularly antioxidants, are crucial for early embryonic development during *in vitro* embryo production. These antioxidants help combat the effects of ROS, which can increase significantly under normal culture conditions or during heat stress. The results of this study can aid in the formulation of culture media containing curcumin, which could be beneficial for *in vitro* embryo production under stress conditions. In addition, the study suggests that incorporating turmeric plants as animal feed supplements can help reduce heat stress, facilitating efficient management of breeding programs. This could mitigate economic losses on farms and improve farmers’ incomes, particularly during the summer season.

## Authors’ Contributions

RR: Data curation, formal analysis, investigation, methodology, and writing–original draft, review and editing; SuS: Data curation, writing original draft, investigation, and methodology, SS: data curation, investigation, methodology. PNR: Formal analysis. NLS: formal analysis and writing–original draft. AL: Conceptualization. MKS: Conceptualization, project administration, supervision, review, and editing. All authors have read and approved the final manuscript.
